# The impact of participatory arts in promoting social relationships for older people within care homes

**DOI:** 10.1177/1757913920921204

**Published:** 2020-06-07

**Authors:** A Dadswell, H Bungay, C Wilson, C Munn-Giddings

**Affiliations:** Research Fellow, Faculty of Health, Education, Medicine and Social Care, Anglia Ruskin University, Bishop Hall Lane, Chelmsford CM1 1SQ, UK; Reader in Health Services Research, Faculty of Health, Education, Medicine and Social Care, Anglia Ruskin University, Cambridge, UK; Senior Research Fellow, Faculty of Health, Education, Medicine and Social Care, Anglia Ruskin University, Chelmsford, UK; Professor of Participative Inquiry and Collaborative Practice, Faculty of Health, Education, Medicine and Social Care, Anglia Ruskin University, Chelmsford, UK

**Keywords:** loneliness, older people, participatory arts, social isolation, social relationships

## Abstract

**Aims:**

Loneliness and social isolation negatively affect wellbeing and quality of life. Despite the proximity of others, older people living in care homes often experience loneliness and social isolation. The impact of participatory arts on wellbeing is widely acknowledged; however, relational impacts have received less attention. This article explores the impact of participatory arts in care homes on the social relationships between older people and older people and care staff.

**Methods:**

‘Creative Journeys’, an initiative led by Essex County Council, provides opportunities for older people living in care homes to participate in arts activities. In this study, three arts organisations (reminiscence arts, seated dance, and orchestral music participation) delivered participatory arts in three homes. Stage 1 of the research comprised mixed-methods case studies in each home. Stage 2 involved an online survey across care homes in Essex to provide a broader perspective, with follow-up interviews in three further homes, and a focus group with the arts organisations. Findings presented here focus on the qualitative data around the impact of participatory arts on the social relationships in care homes between older people and older people and care staff.

**Results:**

Participatory arts enhanced social relationships between older people and between older people and care staff in care homes. Through engagement in shared experiences leading to increased communication and interaction, participatory arts facilitated social connectedness between residents, and changed the relationship dynamics between older people and care staff, thus promoting reciprocity.

**Conclusion:**

Participatory arts enable older people to express themselves creatively, and make meaningful contributions to their social relationships. Policy makers and those working in the care sector should consider including participatory arts as an integral and necessary component of quality care for older people living in care homes.

## Introduction

When older people move into residential care homes, they often experience loneliness and social isolation. Loneliness is a subjective experience with feelings of emptiness and rejection,^1^ due to the number of relationships a person has, or the quality of those relationships not meeting their expectations.^2^ In contrast, social isolation is an objective circumstance where a person has a small social network, or is separated from their network.^1^ Loneliness and social isolation are clearly linked, but do not always present together; for example, a person may be isolated due to a small network but protected against loneliness by a few deep and meaningful relationships.^3^ The direct consequences of loneliness are both social and emotional,^4,5^ but loneliness and social isolation can also be detrimental to health, wellbeing and quality of life.^6^ A recent literature review found the most prevalent health outcome of loneliness in old age was depression, while for social isolation it was cardiovascular problems.^7^ In 2018, the UK Government launched the first Loneliness Strategy,^8^ which implicitly addresses aspects of social isolation too. Similarly, the Campaign to End Loneliness aims to tackle loneliness and social isolation particularly in older people.^9^

As people age, they may be more susceptible to experiencing loneliness and social isolation due to the loss of social networks through life transitions such as retirement, bereavement, reduced mobility and health deterioration.^6^ Moving into a care home marks another life transition. Currently, 4% of those aged above 65 years and 16% of those aged above 85 years are living in care homes in the UK.^10^ Despite the proximity of others, people living in care homes are twice as likely to experience loneliness compared with people living in the community.^11^ Reasons for moving to a care home include poor physical health and/or cognitive impairments, which can create barriers to interaction between residents,^12^ and establishing meaningful interpersonal relationships can be difficult.^13,14^ Previous research has explored the importance of building relationships in care homes, and the different types of relationships that can develop;^15–17^ the current research turns attention towards how these relationships can develop and be supported.

The impact of participatory arts on the wellbeing of older people is widely acknowledged.^18,19^ The Creative Health Report^20^ states that the arts can ‘help meet major challenges facing health and social care’ (p. 4) including ageing, loneliness and social isolation. However, there is little research that investigates how participatory arts may promote social relationships and therefore reduce loneliness and social isolation. A recent review of the literature identified that the arts promoted a sense of belonging to an artistic community and contentment with social lives, addressing discrepancies between desired relationships and reality.^3^ Arts also facilitated interactions and enhanced social capital, helping to strengthen existing relationships and build new ones. Finally, arts enabled older people of all capacities to engage in meaningful social participation and make a valued contribution to their relationships and communities, indicating the potential of participatory arts to alleviate loneliness and social isolation experienced in care homes.

Introducing participatory arts in care homes presents an opportunity for residents to engage in ‘meaningful activities’, as recommended by the National Institute of Health and Care Excellence.^21^ In terms of social relationships, it has been found that participatory arts programmes provided opportunities for meaningful social contact, support and friendship, improved relationships between people living in care homes, and fostered a better sense of social cohesion and community for those with dementia in care homes.^22^ More recently, outcomes of improved communication, increased socialisation, and a better atmosphere have been identified.^23^ These findings are significant given the central importance of relationships in the lives of residents.^13,24,25^ However, none of these studies focused specifically on social relationships or social wellbeing, and most considered only one art-form. In contrast, the study reported here explored the impact of participatory arts across different art-forms on the social relationships between older people, and between older people and care home staff, within Essex care homes.

## Methods

‘Creative Journeys’ is an initiative led by Essex County Council’s (ECC) Culture and Community Engagement Team providing opportunities for older people living in care homes to participate in a range of arts activities. Research undertaken by Anglia Ruskin University (ARU) and the Older People’s Research Group, Essex (OPRGE), an independent research group led by and for older people, aimed to generate evidence for the impact as well as the mechanisms through which participatory arts can build social relationships and address issues of loneliness and social isolation in care homes. Ethical approval was received from ECC’s Research Governance Group on 30 December 2016 and ARU’s Faculty Research Ethics Panel. Members of the OPRGE contributed to each stage of the research. This article presents the qualitative findings from the interviews, focus groups, structured and unstructured observations and free-text survey comments across the two stages of the research. The full report is available elsewhere.^26^

### Stage 1

Stage 1 of the research comprised case studies conducted in three care homes in Essex. Three arts organisations delivered a programme of activities in one of the three homes. Age exchange used reminiscence arts, theatre and story-telling to explore memories, stories and experiences. Activities varied, but often involved introducing an object/artefact to spark conversation and story-telling. They worked with eight female residents along with the activities coordinator for 2 h each week over 10 weeks, with the final two sessions lasting longer to facilitate filming the residents telling their stories. This care home had 106 beds across three floors that functioned as separate units. Residents who took part were from different units and were particularly frail, with specific physical/cognitive impairments which meant that initial plans to create a piece of theatre had to be adapted. Green Candle Dance Company facilitated seated dance activities for 12–20 female residents who took part in 2-h sessions each week for 12 weeks. Each session took a different theme that would determine the props and guide dance moves, such as the seaside. A pianist or guitarist would follow the flow of the sessions and provide live music accompaniment. A total of 54 residents lived in this home, 29 in the residential unit and 25 in the dementia unit. Those who took part were from both units and had a range of capacities and conditions, and were supported by two activities coordinators, a staff member who worked in the laundry and a family member who attended every week. Sinfonia Viva with Orchestras Live delivered orchestral experiences including activities such as residents singing, conducting and writing their own song. Around 20 residents were involved at different points over three half-day sessions that culminated in a final performance in a local venue. A professional orchestral composer and cellist led the sessions, with support from a trumpeter, a violinist and an education officer. The care home had a total of 47 residents, many of whom had dementia to varying degrees. Two activities coordinators supported the programme, and many staff members, volunteers and family members helped with and attended the final performance.

Data collection methods included quantitative wellbeing and social relationship measures^26^ observations, semi-structured interviews and focus groups with residents, relatives, care home staff and arts facilitators. Observations were undertaken by the first and third authors, as well as a member of the OPRGE at four full sessions in each of the homes (*n* = 12). A bespoke structured observation tool, the Social Interaction in Residential Settings (SIRS) observation schedule (see Supplemental material), was developed to capture the nature of the social interactions that occurred and what activities/actions/events preceded the interactions. Meanwhile unstructured observations captured wider contextual details. Observations were of all the residents, staff members and artists taking part in the sessions. Semi-structured interviews and focus groups were conducted by the first and third authors at the end of each programme with residents, care home managers, activities coordinators, care home staff, relatives and arts facilitators. They explored the impacts of the activities on social interaction and relationships, as well as perceived enablers and barriers. An example of one of the questions we asked the residents is: ‘Was there anything about the arts programme that helped you to engage with other residents/staff in the care home?’ and for activities coordinators: ‘Have you noticed any changes in the relationships between residents and/or residents and staff?’ Follow-up interviews with residents, activities coordinators and care home staff took place 3 months later to gauge the development and sustainability of impacts. Unfortunately, one of the care homes closed before the follow-up interviews took place. Across the case studies, we conducted interviews/focus groups with 20 residents, 12 care home staff and two relatives.

### Stage 2

Stage 2 included an online survey to provide a broader perspective of the use of arts activities in residential care homes across Essex. Stage 1 data informed the survey development, with a total of 24 questions. These included a number of free-text questions to identify enablers and barriers to providing arts activities, perceived immediate and long-term impacts, and any particular qualities of arts and arts facilitators considered to promote those impacts. A total of 27 care homes took part in the survey, with 13 managers, 11 activities coordinators (or equivalent), one senior carer, one team leader and one admin manager completing the survey on behalf of their respective homes. Three care homes who completed the survey were invited to take part in follow-up interviews to explore the issues raised in more depth. Semi-structured interviews were conducted by the first and third authors with seven residents and 12 staff members (care home managers, activities coordinators and carers). Survey topics included what arts activities take place and who delivers them; what is the motivation behind delivering or participating in arts activities; and the effect they have on residents, staff; and connections with the local community, with a particular focus on social relationships. An example interview question asked of the residents is: ‘Do you think there’s anything special about arts activities that makes a difference to your relationships with other people?’ and for care home managers: ‘Can you tell me about whether you think the arts activities could be used to help to connect residents with the local community?’ Finally, a focus group conducted by the second author took place with five representatives from the stage 1 arts organisations, exploring their shared learning, perceptions of the distinctive features of the art-forms that helped to build relationships, and perceived enablers and barriers to the impacts of the arts on social relationships.

The multiple data collection methods informed the overall understanding of how the arts impacted on social relationships in care homes, allowing for a more holistic view of the impacts and the mechanisms through which participatory arts can build social relationships.

### Data analysis

The data from the interviews, focus groups, observations and free-text survey comments were subjected to thematic analysis following Braun and Clarke.^27^ Group data analysis sessions (with ARU researchers and members of the OPRGE) enabled us to reach consensus on themes, and ensured rigour and transparency. Initial analysis was conducted after completion of the stage 1 data collection, with the research team individually reading through interview/focus group transcripts and observation notes and making notes about potential codes, followed by a group meeting to agree codes and collate themes. The first and third authors then re-read transcripts/observation notes to refine the themes and ensure key points in the data had been captured. All authors reviewed themes and agreed theme labels. Initial analysis then informed our survey, interview and focus group questions in stage 2. The same data analysis approach was followed for stage 2 data. The authors then reviewed the analysis from stages 1 and 2 and identified themes which cut across both stages (and across each method of data collection). Due to the similarities in findings across stages and data collection methods, it was deemed most appropriate to present themes from across both stages of the research. The findings below bring together data from across the different qualitative data collection methods to look at the impact of arts on the social relationships of older people in care homes.

## Results

The data showed that involvement in participatory arts helped to address issues of loneliness and social isolation by promoting the social relationships between older people and between older people and staff in care homes. Four overarching themes were identified: engagement in shared experiences, communication and interaction, social connectedness and changing dynamics of relationships. A number of mediating factors that could be enablers or barriers to the impact of participatory arts on social relationships were also identified, including factors relating to the care context, such as the care home capacity and culture, the creative process itself, and the approach of the arts facilitator, which are described elsewhere.^26^

### Engagement in shared experiences

Social relationships were facilitated through bringing people together for a shared experience, including residents who did not usually participate in group activities:

*[I]t brings together residents that would not normally choose to do other activities and builds friendships within the home.* (Survey respondent)

*It was nice to join with people, that perhaps you didn’t see all that often.* (Stage 1 resident)

*You meet more people, like, intimately, than you would normally …* (Stage 1 resident)

*One particular resident stood out as being someone that we’ve not really had great success in engaging with a group. He’s a very solitary person, and has always, happily, done things on his own, and doesn’t join in overly in a group. From the moment it started, he responded with both the music and the singing, and the actions actually. He was someone that definitely, we saw a huge difference in his way of being.* (Stage 1 care home manager)

Residents and staff were able to engage with each other and to a greater extent than they normally would when doing something creative and having fun together:

*So, it’s nice that they can talk to each other and learn more about each other. Because then it builds stronger friendships.* (Stage 2 staff member)

*Again shared interests, laughing together, having fun together makes a bond stronger, getting to know families and develops easier communication*. (Survey respondent)

Finally, the activities enabled residents and staff to share quality time together, which is not always possible in care homes. These enjoyable shared experiences and quality time also promoted staff satisfaction and morale:

*Yes staff do enjoy the activities. It helps when we have entertainers in as it can free up some time. It can mean the team enjoy an activity with the residents, it makes it a fun place to work.* (Survey respondent)

*I certainly know a lot more people now, know a lot more about them, as well, and have interacted with a lot more than I would have done …* (Stage 1 staff member)

*Spending time with them doing stuff they enjoy makes us happier, to see them actually having a good time, rather than sitting and being quite withdrawn themselves.* (Stage 2 staff member)

### Communication and interaction

Observations of the activities showed that residents expressed themselves verbally but also using non-verbal forms of communication, including facial expressions, smiling and making eye contact, and through modelling or mirroring dance moves and dancing with partners:

*The skills that they picked up over that time were very varied, from the actual motor skills of expression and physical movement, and then, also, very creative skills.* (Stage 1 arts facilitator)

Touch was also observed as a form of non-verbal communication, particularly between care home staff and residents, for example, staff holding hands with residents, helping them to move to the music and hugging or stroking the arm of a resident when they were sharing emotional memories or were unsure of the activity.

In addition, new topics of conversation were initiated during the activities, sometimes about the arts themselves while other times the arts facilitator would encourage everyone to share ideas, memories and stories from their lives:

*I saw how it affected people’s relationships when they were talking about the music or the way they’d been encouraged to be involved in the discussions about lyrics and songs or what the actual programmes would involve. For me it was really interesting to see that actually it brought in some conversation to people’s days because they do chat to each other but quite a lot of the time, because of their dementia, it can often be just nonsense really. This was a structured conversation … they were just expressing their views.* (Stage 1 activities coordinator)

This helped all participants get to know each other and build their confidence in interacting with each other. Stage 1 follow-up interviews found that interactions and conversations continued outside of the arts activities, and residents had more confidence to get involved:

*They’ve learnt each other’s ways now. They feel comfortable with each other, so that means that when they go past each other they’ll say, ‘Hello’. They’ll communicate and say, ‘How was your day?’ Rather than just walking by and going, ‘Oh hello’ politely …* (Stage 1 activities coordinator)

*… with one of my ladies, she’s got more confidence to talk to people. And there’s more people getting involved in things now.* (Stage 1 staff member)

*Normally, if I sort of moved around the home, you go past people and you just smile. But, now we stop and say hello. We have a little chat. Yes, it has improved things.* (Stage 1 resident)

### Social connectedness between older people

For residents, participatory arts produced a sense of collective enjoyment. This was demonstrated in the observations and the interviews, with participants not only having a good time together but also feeling happy that other residents were enjoying themselves:

*Enjoying each other’s company, doing something together.* (Stage 1 resident)

*I think it was the general atmosphere. Watching other people really enjoying themselves … I really love it. I really do … It was so nice … seeing the people from the [dementia unit] getting so much out of it.* (Stage 1 resident)

Building on these connections, observations and interviews also showed how the residents supported each other during the activities, for example, by modelling dance moves or encouraging each other to join in:

*If somebody wasn’t sure what they were doing, and they were next to someone who did they really helped each other, which was lovely … Then there was someone like [Gladys], who was, kind of, more encouraging people by being quite enthusiastic. So, she would get up and then other people would be like, ‘Oh, we could do that too’, and so she would encourage people in that more dynamic way.* (Stage 1 arts facilitator)

This suggests how participatory arts can promote reciprocity in relationships within care homes whereby residents are able to make a contribution by helping others. Responses to the survey also indicated how participatory arts provided opportunities for residents to support and praise each other, including those who may not be directly involved in the activity:

*They appear to enjoy compliments within the peer group.* (Survey respondent)

*Our residents’ choir also has a great following from our non-participating residents.* (Survey respondent)

The closeness and camaraderie that developed through the participatory arts built social connections, for example, some residents in stage 1 described their new group identity as being a member of a *‘club’*:

*Yes, it’s made it more a communal feeling.* (Stage 1 activities coordinator)

*We didn’t know each other before but we all felt close [during the activity].* (Stage 1 resident)

*We were like a little family.* (Stage 1 resident)

*I think they really interacted really, really well with each other, which created a very fantastic group dynamic. The best kinds of group sessions are always when the group take off … and that camaraderie … They listen to each other’s stories.* (Stage 1 arts facilitator)

Residents developed new friendships or strengthened existing ones, and this led to a sense of community:

*[Margaret] was a bit down because she was new here, but she’s got friends now. She’s made friends. She’s got a best friend in [Mary] now because of [the activities].* (Stage 1 staff member)

*I think coming together on those sessions, and then finally with the end result … I think that definitely benefitted with certain friendships, because friendships have grown here. Different people, friendship groups, were being stronger because of it.* (Stage 1 care manager)

### Changing dynamics between older people and care home staff

By participating in something new and different together, residents and care home staff were on a more equal footing, which changed the dynamics of their relationships. Observations and interviews suggested that residents could relate to the artistic material presented to them – such as familiar songs or old objects – and shared memories and knowledge of these things in the group, while care home staff were often unfamiliar with such history:

*Oh yes. Oh anything where I knew the words, yes.* (Stage 1 resident)

*They’ve [care staff] probably listened to Radio 1 their whole lives and then all of a sudden along comes classical music and it just brings a different power and emotion to it.* (Stage 1 activities coordinator)

*… never heard anything like that before.* (Stage 1 staff member)

Residents were empowered to take on different roles within the group, for example, being the lead storyteller, dance instructor or music conductor, which changed the usual dynamic where care home staff lead day-to-day activities. It also helped staff and artists to see residents in a new light:

*So, it was quite nice to see some of the more creative ones having an opportunity to be quite free and to be the leader. Because, actually, some of the people with more advanced dementia were actually more creative. So, to give them that time to lead and to feel … a sense of ownership, I guess, was really nice.* (Stage 1 arts facilitator)

*It’s taught me a lot. It showed me a lot about my residents …* (Stage 1 staff member)

*She looks so disabled you think ‘Is she just going to sit in the corner all the time like that, but listen and maybe enjoy it?’ It was amazing that she turned out one of the lead storytellers, the most lively in terms of her engagement.* (Stage 1 arts facilitator)

In addition, residents became more confident in talking with care home staff. The activities enabled more informal and personal interactions. This was not limited to the staff that participated in the arts sessions, but also other staff who would ask residents about what they had been doing:

*She’s an open book now, she doesn’t stop … she’s got the confidence to talk about things, now, and ask and say, ‘Well, actually I don’t like cornflakes. I want porridge, and I need someone to help me eat it because I can’t’.* (Stage 1 activities coordinator)

*Well yes, the friendly talk, you know, about our lives as well as theirs. Yes. No. that was very good. I enjoyed it anyway.* (Stage 1 resident)

*I think for a start it can give us something to talk about to [the staff], because obviously the staff can’t be here, but I think generally speaking, well let’s say the bonding. It helps, definitely.* (Stage 1 resident)

Finally, through involvement in participatory arts and watching the arts facilitators, care home staff learnt new skills and techniques for engaging with residents:

*I learnt new interaction techniques, techniques to get a group of people able to communicate with each other more as well …* (Stage 1 staff member)

*You just bring up bits and pieces about what they have mentioned about the past, and that is when they start feeling comfortable again, and then they will have a laugh and joke with you. It has brought a few of them out of themselves.* (Stage 1 staff member)

## Discussion

The findings demonstrate the impact of participatory arts on the social relationships between older people and between older people and staff in care homes, with the potential to address issues of loneliness and social isolation. Engagement in shared experiences leading to increased communication and interaction has the potential to address social isolation in particular. Previous research reported that residents found it difficult to make friends and foster intimate relationships with other residents because they had no apparent common interests, and the ability to connect was even more difficult when residents had different levels of cognition.^13^ Our findings, however, showed that the shared experience of participatory arts, promoted interactions within sessions and provided new topics of conversation for ongoing interactions. In addition, residents with cognitive impairments were able to express themselves through the arts, which may make it easier for them to engage with others. In terms of relationships between residents and staff, participatory arts allowed opportunities to share meaningful experiences and spend time together beyond care routines. Indeed, spending ‘non-care’ time together is crucial for residents to develop close friendships with staff.^25^

Social connectedness is a fundamental human need, and can be a protective factor against loneliness.^24^ Our findings show that participatory arts promoted social connectedness through facilitating collective enjoyment, supporting and encouraging others, developing a sense of camaraderie and community and strengthening friendships. These impacts demonstrated reciprocity whereby residents were able to make a meaningful contribution to the group or relationship; an important factor in older people’s relationships and wellbeing.^28^ Residents are otherwise unlikely to be engaged in reciprocal relationships,^29^ and can experience loneliness and a loss of meaning and identity as they transition to becoming largely cared for.^30^

Reciprocity was also observed in the changing dynamics between residents and care home staff. Residents shared knowledge and memories of objects and histories that staff had little awareness of and took on lead roles during arts activities, allowing staff to see them in a new light and value their contribution. Indeed, it has been suggested that reciprocity in caring relationships empowers not only the resident but also the carer, and benefits the whole care home community.^31^ Furthermore, through the participatory arts, residents gained confidence, staff learnt new skills to engage residents, and both felt more comfortable to interact informally and on a personal level with each other. This is significant, as it has been suggested that relationships with staff are hugely important in addressing the loneliness and social isolation of residents and may become a substitute for their lost relationships with friends and family outside of the care home.^13^

Our findings clearly indicate the potential of participatory arts to respond to issues of loneliness and social isolation particularly in care homes. Despite this, the UK Government Loneliness Strategy^8^ only mentions the arts as part of broader social prescribing schemes, which are unlikely to be inclusive of care homes. Similarly, in the Age UK and Campaign to End Loneliness report on approaches to reduce loneliness and isolation in later life,^32^ creative approaches using the arts are only briefly referred to as part of group approaches that focus predominantly on coffee mornings and non-arts-based activities. The report identifies a knowledge gap in our understanding of loneliness and social isolation in care homes. Indeed, people living in care homes are often excluded from the discourse on loneliness and social isolation, evident in a recent analysis of characteristics and circumstances associated with loneliness in England,^33^ which was based on data from people living in the community.

Though this research contributes evidence on the potential of participatory arts in tackling loneliness and social isolation in care homes, there were a number of challenges and limitations to the design. Challenges were centred largely around care home capacity and routines, and in conducting the research in the care context; for example, our methods did not include an established measure of loneliness or social isolation in part due to the challenges of employing established baseline and end-point measures with care home residents. Also, it would have been useful to have captured the views of residents and staff who had not participated in the activity and may have expressed negative views about the project. The arts organisations in the focus group also described instances in other care homes where staff had been unsupportive of the sessions although this was not observed in the case studies.

## Conclusion

Participatory arts engage older people, enable them to express themselves creatively, connect with others including care home staff and provide the opportunity for them to make meaningful contributions to their social relationships in care homes. To implement this in practice we recommend training opportunities for care home staff, and particularly activities coordinators, in delivering participatory arts for their residents. This could be delivered as part of arts programmes from external arts organisations, who should aim to leave a lasting legacy through their work to promote sustainability. Furthermore, policy makers and those working in the care sector should consider including participatory arts as an integral and necessary component of quality care for older people living in care homes. Further research into this area could look specifically at older people’s feelings of social connectedness and reciprocal relationships within care homes, and how participatory arts can specifically target, encourage and promote these to address loneliness and social isolation.

## Supplemental Material

Supplemental_material_-_SIRS – Supplemental material for The impact of participatory arts in promoting social relationships for older people within care homes Click here for additional data file. Supplemental material, Supplemental_material_-_SIRS for The impact of participatory arts in promoting social relationships for older people within care homes by A Dadswell, H Bungay, C Wilson and C Munn-Giddings in Perspectives in Public Health
