# Supplementary material for: The impact of participatory arts in promoting social relationships for older people within care homes
Source: Perspect Public Health. 2020 Jun 7;140(5):286–93. doi: 10.1177/1757913920921204 (PMC7522769; doi:10.1177/1757913920921204)
Supplement: Supplemental_material_-_SIRS – Supplemental material for The impact of participatory arts in promoting social relationships for older people within care homes [file Supplemental_material_-_SIRS.pdf]

Supplemental material

Social **I**nteraction in **R**esidential **S**ettings (SIRS) Observation Schedule

XXX, XXX, XXX and XXX (2019)

Location: ___________________ Activity/organisation: ______________________

Date: ________ Start time: _____ Session number: ____ Observer initials: ____

Total number of residents: ________ Total number of staff: ________

Key: Verbal = V Non-verbal = NV Residents = R Residents & staff = RS

**Social Interaction in Residential Settings (SIRS) Observation Schedule**					
**No.**	**Nature of interaction**	**Trigger**	**Verbal / non - verbal**	**Residents / residents & staff**	**No. of residents**
E.g.	*Residents talk with one another about dancing to this music in their younger years*	*Facilitator plays an old piece of music to the group*	*V*	*R*	*5*
1					
2					
3					
4					
5					
6					
7					
8					
9					
10					

11					
12					
13					
14					
15					
16					
17					
18					
19					
20					
21					
22					
23					
24					
25					
